# Special Issue: Phage–Bacteria Interplay in Health and Disease

**DOI:** 10.3390/v14051054

**Published:** 2022-05-16

**Authors:** Zuzanna Drulis-Kawa, Daria Augustyniak

**Affiliations:** Department of Pathogen Biology and Immunology, Faculty of Biological Sciences, University of Wroclaw, Przybyszewskiego 63/77, 51-148 Wroclaw, Poland

Bacteriophages are obligatory parasites propagating in bacterial hosts in a lytic or lysogenic/pseudolysogenic cycle. Phages are the most abundant biological particles in the world, being responsible for: (i) dissolved and particulate organic matter circulation via host cell lysis, (ii) regulation of numbers and biodiversity of populations, (iii) horizontal gene transfer (HGT) via transduction, or indirectly via transformation of bacterial DNA released during cell lysis, and, finally, (iv) lysogenic conversion by temperate phages. Therefore, phages greatly affect microbial diversification as an integral part of each ecological niche, including the human body. The tremendous dynamics of phage–host interactions result in the continuous flow of genetic material, which drives the co-evolution of both entities.

Advances in recent years in molecular biology, multi-omics, and bioinformatic approaches have contributed to the deeper insight into and functional analyses of a wide range of phage–bacteria interactions at the individual and population levels, and their consequences on health and disease. The Special Issue of *Viruses* “Phage–Bacteria Interplay in Health and Disease” focuses primarily on the regulation and functioning of human/mammal microbial ecosystems as the consequence of specific and non-specific virus–bacteria interactions, bacterial defence against phages which can drive the outcome of the disease/infection, phages as human immunomodulators, and the description of innovative experimental techniques characterizing the phage traces in mammals.

A total of 13 manuscripts, including 10 original and 3 review papers, have been published in this Special Issue. The collection contains recent papers that could be roughly classified into three themes comprising the following: (1) survival strategies of bacteria in response to phage infection as well as the impact of phages on bacterial virulence and pathogenicity (theme 1, five papers); (2) influence of lytic and temperate phages on the human microbiome as well as human virome composition (theme 2, three papers); (3) phages as potential antibacterial therapeutics (theme 3, five papers). All papers reflect the impact of phage–bacteria as well as phage–bacteria–host interactions on human health, disease, and economic activities. They also highlight the great importance and relevance of the topic addressed.

*Streptococcus pyogenes* is a Gram-positive β-hemolytic pathogen that strictly infects humans and can cause superficial skin infections, pharyngitis, toxin-mediated diseases (e.g., streptococcal toxic shock syndrome (STSS)), and invasive diseases of subcutaneous tissues [1]. Bacteria have developed different defence strategies against phage infection ranging from adsorption prevention to a wide panel of intracellular mechanisms [2]. Beerens et al. [1] described several mechanisms utilized by *S. pyogenes* to protect against Phage A1. Phage A1, as a temperate phage, was able to infect *S. pyogenes* strains, resulting in complete resistance against subsequent phage infections, most likely by providing superinfection immunity. Furthermore, the lysogenization did not influence the humoral host immune response or bacterial virulence and did not induce ampicillin tolerance to this common antistreptococcal antibiotic. The authors demonstrated that the type II-A CRISPR-Cas system of *S. pyogenes* acquires new spacers upon phage infection, which are increasingly detectable in the absence of a capsule. Finally, the authors showed that the number of infecting phages is limited through binding to released streptococcal outer membrane vesicles. The authors proposed a multistage interaction model between *S. pyogenes* and Phage A1 [1].

Outer membrane vesicles (OMVs) of Gram-negative bacteria are important virulence factors as decoys against a variety of antibacterials but are also an element in host–bacteria interplay, and intra- or inter-species bacterial communication [3,4]. In the paper of Augustyniak et al. [5], the authors studied the *Pseudomonas aeruginosa* antiviral strategy based on outer membrane vesicles (OMVs) against the LPS-specific phages KT28 and LUZ7. To investigate the passive and active role played by OMVs towards these phages, the OMVs derived from the phage-sensitive wild-type PAO1 strain, and an LPS-deficient mutant (*∆wbpl* PAO1) resistant to both phages, were used. It turned out that naturally formed OMVs efficiently protected *P. aeruginosa* from phage infection. Next, it was verified whether OMVs derived from the wild-type PAO1 strain were able to sensitize the LPS-deficient mutant (*Δwbpl* PAO1) to the tested phages. The growth kinetic curves and one-step growth assay revealed no sensitization event [5].

*Clostridioides difficile* is one of the most common causes of antibiotic-related nosocomial infections with symptoms ranging from mild diarrhea to life-threatening pseudomembranous colitis and/or toxic megacolon [6]. Therefore, one work explored the therapeutic potential of the *C. difficile* phages, with a particular interest in phage CDHS-1 propagating on the hypervirulent 027 ribotype *C. difficile* R20291. The study focused on the transcriptomic takeover of phage CDHS-1 during infection, and analyses revealed that the majority of the bacterial genes connected with metabolism and toxin production were downregulated at the early phase of CDHS-1 replication, whereas genes related to DNA synthesis and ATP production were among those upregulated at this stage. The holin, endolysin, and structural genes were upregulated towards the mid-log and late phases of the phage replication. The retrieved phage-resistant clones and lysogens showed relatively low virulence in the larval model of *Galleria mellonella* compared to the wild-type strain. The data suggest that phage infection both reduces bacterial colonization and negatively impacts bacterial pathogenicity, supporting the therapeutic potential of the phage for human and animal use [6].

Topka-Bielecka et al. [7] described the characterization of phage vB_EfaS-271 specific to *Enterococcus faecalis* strains. The report indicated that vB_EfaS-271 can significantly decrease the number of viable *E. faecalis* cells in biofilms formed on catheters and in liquid cultures and revealed no considerable toxicity to mammalian cells, influencing neither their viability nor morphology. The efficiency of phage resistance development was especially significant under conditions of high MOI values; nevertheless, phage vB_EfaS-271 was considered promising for phage therapy [7].

Lichvariková et al. [8] performed genome analyses of the prophage content in *Streptococcus agalactiae* (GBS) clinical isolates to verify the prevalence of prophages versus the bacterial virulence potential. Based on the whole genome sequencing and PCR methods, the authors identified eight groups of prophages, amongst which the highest prevalence was observed for a prophage from group A (71%) and a satellite prophage from group B (62%). They observed that the prophage distribution did not differ between clinical and screening strains, but it was unevenly distributed in MLST (multilocus sequence typing) sequence types. This study implies that prophages could be beneficial for the host bacterium [8].

Phages have an important role in shaping bacterial communities. Phages also impact human health by infecting bacteria forming human microbiomes in the gut, respiratory tract, skin, or vagina. Happel et al. [9] investigated the prevalence and persistence of (pro)phages and their associations with the vaginal bacterial community composition in the female genital tract of 13 South African adolescents, who received no antibiotics. The shotgun metagenome sequencing of cervicovaginal samples collected longitudinally showed that the most prevalent phage family was *Siphoviridae*, followed by *Myoviridae*, *Podoviridae*, *Herelleviridae*, and *Inoviridae*. The results pointed out that some prophages were present in cervicovaginal secretions of multiple participants, suggesting that prophages, and thus bacterial strains, are shared between adolescents. Shaping the biodiversity of bacterial populations, these viruses contribute to local fluctuations in the vaginal microbiome [9].

Bacterial vaginosis (BV) is characterized by a reduction in *Lactobacillus* (L.) spp. abundance and increased colonization of facultative anaerobes, such as *Gardnerella* spp. [10]. The paper of Jacobsen et al. [11] related to vaginal microbiota, studying the role of phages in the perturbation of the vaginal bacterial community. Vaginal samples from 48 patients who underwent in vitro fertilization treatment for non-female factor infertility were subjected to metagenomic analysis of purified virus-like particles. It turned out that the vaginal virome was linked with the BV status and bacterial community structure. The viral community structure was strongly correlated with the presence of key beneficial bacterial species (*L. crispatus* and *L. iners*) as well as with known pathobionts (*Gardnerella* spp. and *Atopobium vaginae*) [11].

Mayne et al. [12] investigated the influence of an anti-salmonella phage preparation (BAFASAL^®^) on the ex vivo human gut microbiome composition and function. Using a novel in vitro assay called RapidAIM as well as 16S rRNA gene sequencing and metaproteomic approaches, the authors documented that the ex vivo human gut microbiota composition and function were unaffected by BAFASAL^®^ treatment, which proves the GRAS provision (generally recognized as safe) [12].

At present, traditional antimicrobials are becoming ineffective against multidrug-resistant bacterial pathogens. Therefore, phages are increasingly beginning to be recognized as an alternative or supportive therapeutic agent [13].

Shimamori et al. [14] investigated the efficacy of the *Staphylococcus aureus* phage in the treatment of atopic dermatitis (AD), the most common inflammatory skin disease, in an atopic mouse model. As previously documented, phage SaGU1 can infect a broad range of *S. aureus* in AD patients, whereas it does not kill strains of the symbiotic bacterium *S. epidermidis*. In this work, the authors showed that administration of SaGU1 to the back skin of mice reduced both *S. aureus* counts and the disease exacerbation caused by these bacteria. Furthermore, the application of *S. epidermidis* in combination with SaGU1 inhibited the emergence of phage-resistant *S. aureus*, indicating that synergistic use of probiotics and phages can be promising and effective in the treatment of *S. aureus*-associated AD [14].

Since interactions between phages and mammals strongly affect the possible applications of phages, tools to study how phages circulate in the body and can be deposited in tissues are highly desirable. Understanding this need, Kaźmierczak et al. [15] proposed red fluorescent protein (RFP)-labeled *E. coli* lytic phages as a new tool for the investigation of phage interactions with cells and tissues. The deposition of an RFP-displaying phage in multiple murine organs ex vivo after various routes (intravenous, oral, rectal) of phage administration was verified. The most effective intravenous administration led to effective distribution kinetics with phage presence in the lymph nodes, lungs, and liver after 20 min, whereas in the muscles and spleen/lymph nodes, this occurred 30 min and 1 h after administration, respectively [15].

Iszatt et al. [16] provided a thorough review of therapies against widespread or emerging multidrug-resistant bacterial pathogens in respiratory medicine, the challenges faced in generating preclinical data, in vitro and in vivo respiratory models using phages, and, finally, clinical trials of phage therapy in respiratory infection treatment such as acute cystic fibrosis or chronic lung infections [16].

An interesting review of Al Ishaq et al. [17] addressed the issues concerning the efficacy of phages against WHO priority pathogens. A comprehensive literature search was conducted in the EMBASE, Web of Science, and PubMed databases for articles published from 2010 to 2020. This systematic review critically evaluated and summarized 29 published articles on phages as a treatment option against *S. aureus*, *Klebsiella pneumoniae*, *P. aeruginosa*, and *E. faecalis* infection models. It also illustrated appropriate phage selection criteria, as well as recommendations for successful therapy. The main conclusions from the analysis of the selected literature were that phage applications (single or in cocktails) are effective and safe, can work synergistically in combination with certain antibiotics, and may induce the emergence of phage resistance [17].

In parallel with attempts to use phages against nosocomial human pathogens, there has been much work conducted on the use of these viruses as alternative methods to control zoonotic and foodborne pathogens. This problem was addressed in a review paper written by Alomari et al. [18]. The authors discussed commercial phage products used in the biocontrol of foodborne pathogens in various foods as well as the role of phages in food protection and sanitation procedures. Central attention was given to phage efficacy in experimental models of infections with foodborne pathogens, including *Salmonella* spp., *E. coli*, *Campylobacter* spp., *Vibro* spp., and *P. aeruginosa*, and other pathogens, such as *Staphylococcus* spp., *Streptococcus* spp., *Klebsiella* spp., *Acinetobacter* spp., and *Mycobacterium* sp. The varied therapeutic and immunomodulatory activities of phages on humoral and cellular immune response mechanisms were also discussed [18].
